# Selectivity of Pesticides used in Integrated Apple Production to the Lacewing, *Chrysoperla externa*

**DOI:** 10.1673/031.010.12101

**Published:** 2010-07-30

**Authors:** Alexandre Pinho Moura, Geraldo Andrade Carvalho, Valéria Fonseca Moscardini, Olinto Lasmar, Denise Tourino Rezende, Márcio Candeias Marques

**Affiliations:** ^1^ Department of Entomology and Phytopathology/Institute of Biology, Universidade Federal Rural do Rio de Janeiro-UFRRJ, 23890-000 Seropédica, Rio de Janeiro, Brazil; ^2^ Department of Entomology, Universidade Federal de Lavras-UFLA, P.O. Box 3037, 37200-000 Lavras, Minas Gerais, Brazil; ^3^ Escola Politécnica de Saúde Joaquim Venâncio, Fundação Oswaldo Cruz-FIOCRUZ, 21045-900, Av. Brasil, 4365, Manguinhos, Rio de Janeiro, Rio de Janeiro, Brazil

**Keywords:** biological control agents, green lacewings, natural enemies, scanning electronic microscopy

## Abstract

This research aimed to assess the toxicity of the pesticides abamectin 18 CE (0.02 g a.i. L^-1^), carbaryl 480 SC (1.73 g a.i. L^-1^), sulfur 800 GrDA (4.8 g a.i. L^-1^), fenitrothion 500 CE (0.75 g a.i. L^-1^), methidathion 400 CE (0.4 g a.i. L^-1^), and trichlorfon 500 SC (1.5 g a.i. L^-1^) as applied in integrated apple production in Brazil on the survival, oviposition capacity, and egg viability of the lacewing, *Chrysoperla externa* (Hagen) (Neuroptera: Chrysopidae) from Bento Gonçalves and Vacaria, Rio Grande do Sul State, Brazil. An attempt was made to study morphological changes caused by some of these chemicals, by means of ultrastructural analysis, using a scanning electronic microscope. Carbaryl, fenitrothion, and methidathion caused 100% adult mortality for both populations, avoiding evaluation of pesticides' effects on predator reproductive parameters. Abamectin and sulfur also affected the survival of these individuals with mortality rates of 10% and 6.7%, respectively, for adults from Bento Gonçalves, and were harmless to those from Vacaria at the end of evaluation. Trichlorfon was also harmless to adults from both populations. No compound reduced oviposition capacity. *C. externa* from Vacaria presented higher reproductive potential than those from Bento Gonçalves. In relation to egg viability, sulfur was the most damaging compound to both populations of *C. externa*. Ultrastructural analyses showed morphological changes in the micropyle and the chorion of eggs laid by *C. externa* treated with either abamectin or sulfur. The treatment may have influenced the fertilization of *C. externa* eggs and embryonic development. Sulfur was responsible for malformations in the end region of the abdomen and genitals of treated females. When applied to adults, abamectin, sulfur, and trichlorfon were harmless, while carbaryl, fenitrothion, and methidathion were harmful, according to the IOBC classification.

## Introduction

The apple tree was introduced to Brazil in the 1960s in Fraiburgo, Santa Catarina. Since this crop was introduced in the country, farmers have faced attacks by several pests, which cause the loss of up to 100% of the harvest (Ribeiro 1999). Currently the apple tree is considered the most important fructiferous tree of temperate climate cultivated in the country; it has great significance in the domestic market and for exports as well (Silva et al. 2007).

Despite its recent cultivation in Brazilian lands, the national pomiculture is not only supplying the domestic market, but is also establishing itself gradually in international trade and European markets. In 2007, Brazil exported about 95,000 tons of apples to the European Union, with Santa Catarina and Rio Grande do Sul as the most productive states, accounting for about 96% of the Brazilian production of this fruit (Agrianual 2008).

However, the imposed exigencies by this and other consuming markets have forced Brazilian producers to adapt to new methods of fruit production, in other words, integrated production. This system permits the production of better-quality fruits, the reduction in pesticide-use, and the possibility of tracking the final product. In integrated production, there are great efforts to control pests by increasing natural factors of mortality using biological agents such as parasitoids, predators, and entomopathogens, with the focus on predators that are able to consume great quantities of prey.

Among the predators, insects belonging to the family Chrysopidae have been considered voracious organisms with strong adaptability to different agroecosystems (Senior and McEwen 2001; Medina et al. 2003; Athan et al. 2004) and are widely distributed throughout the American continents, occurring from the southeast of the United States to the southern region of South America (Albuquerque et al. 1994). Past research has demonstrated that *Chrysoperla externa* (Hagen) (Neuroptera: Chrysopidae) are effective predators of mites on apples (Miszczak and Niemczyk 1978). In Brazil, *C. externa* is one of the most common species of green lacewings found in agricultural crops including the apple tree (Freitas and Penny 2001). *C. externa* feed on harmful arthropodpests of the apple tree, such as the woolly apple aphid *Eriosoma lanigerum*, the green citrus aphid *Aphis citricola*, the San Jose scale *Quadraspidiotus perniciosus*, and the European red mite *Panonychus ulmi* (Ribeiro 1999).

In this context, the use of selective pesticides, which control pests without affecting the populations of natural enemies in a negative way, constitute an important strategy in the integrated management of pests (Moura and Rocha 2006). It is important to identify and develop selective products and to determine the factors that affect behavior, development, and reproduction of beneficial organisms in a way that can be used in conjunction with biological methods of pest control in the apple tree crop.

The objective of this work was to assess the effects of certain pesticides used in integrated apple production in Brazil on the survival and reproduction of adults of *C. externa*, collected in commercial apple orchards in the towns of Bento Gonçalves (29° 10′ 29″ S; 51° 31′ 19″ W) and Vacaria (28° 30′ 44″ S; 50° 56′ 02″ W), both in Rio Grande do Sul, as well as studying possible morphological changes of *C. externa* eggs caused by these chemical agents via ultrastructural analysis using electronic scanning microscopy.

## Materials and Methods

The rearing and maintenance of both populations of *C. externa* was done in a climatic room, at 25 ± 2° C, 70 ± 10% RH, and a photoperiod of 12:12 L:D. Following the techniques described by Auad et al. (2001) they were fed UV-killed eggs of *Anagasta kuehniella* (Zeller) (Lepidoptera: Pyralidae).

### Pesticides

Commercial formulations of abamectin 18 CE (0.02 g a.i. L^-1^), carbaryl 480 SC (1.73 g a.i. L^-1^), sulfur 800 GrDA (4.8 g a.i. L^-1^), fenitrothion 500 CE (0.75 g a.i. L^-1^), methidathion 400 CE (0.4 g a.i. L^-1^), and trichlorfon 500 SC (1.5 g a.i. L^-1^), recommended for use in integrated apple production in Brazil, were used in the bioassays with adults of *C. externa*. The dosage used was the manufacturer's highest recommended rate for controlling pests and diseases in apple trees. Distilled water was used as the control. The application of the evaluated compounds and distilled water over the insects was made using a Potter's tower (Burkard Scientific Ltd., www.burkard.co.uk) regulated at 15 lb pol-2, ensuring the application of 1.65 to 1.89 mg cm-2 of aqueous pesticide solution, according to methodology suggested by IOBC (Sterk et al. 1999; van de Veire et al. 2002).

### Bioassays

Fifteen pairs (each pair constituted by one male and one female) of *C. externa* from each population, with ages from 0 to 24 h obtained from rearing and selected for treatment were anesthetized with CO2 for one min, and then pesticides and distilled water were applied immediately. Although adult male and female *C. externa* are similar in overall size and appearance, they were sexed by looking closely at the ventral surface of the tip of the abdomen using a stereoscopic microscope (40x) as described by Reddy (2002) and Reddy et al. (2004). Males have a small rounded capsule flanked by two small projections, while females have an oval area bounding a longitudinal slit.

After application of pesticides and distilled water, each pair was transferred to a PVC cage (7.5 cm diameter × 8 cm) covered internally with white filter paper, closed in the superior edge with organza type cloth, supported in a plastic tray (40 cm long × 20 cm wide × 10 cm high), and fed every three days with brewer's yeast and honey in the proportion of 1:1 (v/v). The cages were kept in a climatic room, at 25 ± 2° C, 70 ± 10% RH, and a photoperiod of 12:12 L:D. The evaluations took place at 3, 6, 12, 24, 48, 72, 96, and 120 h after application with the goal of determining the mortality rate of the treated *C. externa*.

Six pairs of *C. externa* from each of the studied populations by treatment among the fifteen pairs that received pesticide application were used for the evaluation of effects of the compounds on the reproduction of this species. The evaluations began three days after the applications and continued twice a day with 12 hour intervals until the start of oviposition.

Four consecutive weeks after the start of the oviposition, the number of eggs deposited was counted at three-day intervals. Ninety-six eggs (by treatment) were separated into microtitration plate compartments using a camel hair brush. The plates were closed with a PVC film and kept under controlled conditions until the eggs hatched, when egg viability was evaluated. The oviposition capacity and egg viability of treated *C. externa* pairs were evaluated.

For the evaluation of adult mortality rate, a fully randomized experimental design in a 2 × 7 (two populations of *C. externa* × seven treatments) factorial scheme was used. Five replicates were used, with the experimental plot constituted by three pairs of *C. externa*. For the evaluation of the effects of the compounds on oviposition capacity and egg viability, a fully randomized experimental design with a 2 × 4 factorial scheme (two populations × four treatments) was used. For the oviposition evaluation, six replicates were used, and each plot was constituted by a *C. externa* couple; while in the evaluation of egg viability, eight replicates were used, and the experimental plot was composed of 12 eggs.

### Pesticides classification

The mortality rate of treated adults was corrected by the Abbott's formula (Abbott 1925). The pesticides were then classified based on the reduction of beneficial capacity and mortality caused to the predator using Equation 1, proposed by Vogt (1992).

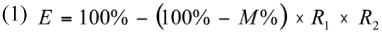

where:

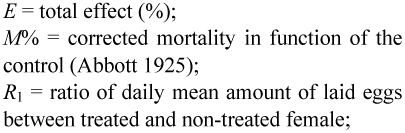


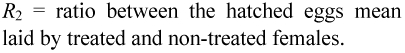

According to recommendations of IOBC, the evaluated pesticides were organized in four toxicological classes (Sterk et al. 1999; van de Veire et al. 2002): class 1 = harmless (E < 30%), class 2 = slightly harmful (30% ≤ E ≤ 80%), class 3 = moderately harmful (80% < E ≤ 99%), and class 4 = harmful (E > 99%).

### Statistical analysis

The obtained data in the bioassays with *C. externa* adults were submitted to analysis of variance using a two-way ANOVA, and the data referring to the number of eggs deposited by female *C. externa* and to the eggs' viability followed a split spot arrangement. The means of the different treatments were compared using the Scott-Knott clustering test (Scott and Knott 1974) at 5% significance when the *F*-test was significant using the statistical software, SAS (SAS Institute 2001).

The mortality data obtained from the bioassays with *C. externa* adults were angular-transformed (arcsine √x/100 transformation) before processing variance analysis. Data about amount of eggs laid per female were transformed to √x+1.

Data referring to the oviposition from females treated with pesticides as well as distilled water (control) were subjected to a model analysis using the software R (R Development Core Team 2006). GLM mode (Generalized Linear Models) with negative binomial distribution of error (logarithmic linkage function) for the over dispersion correction was applied for the output variable of oviposition (Crawley 2002). The following input variables were used to fit the model: *C. externa* populations, time (in days) after oviposition beginning, and treatments. Residual analyses with envelope approach generating probability distribution graphs of Normal (Gauss), Poisson, Binomial, and Negative Binomial (Pascal) were performed to verify how the data fit the models (Paula 2004). The best fitting model choice to oviposition data collected were the graphs plotted by the envelope approach and the AIC index (Akaike Information Criteria) (Akaike 1974; apud Paula 2004), as well as in the relationship between the deviance and degrees of freedom of the residue.

After the choice of the model, the necessary parameters estimates were calculated (Table 1) allowing the oviposition equations to be constructed for both *C. externa* populations and the evaluated treatments. Then, a program was developed to adjust several possibilities of the oviposition predator with all the equations being based on the general one (Equation 2) that follows. This program was implemented through the R software (R Development Core Team 2006).

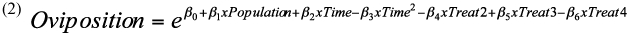

where:

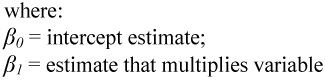

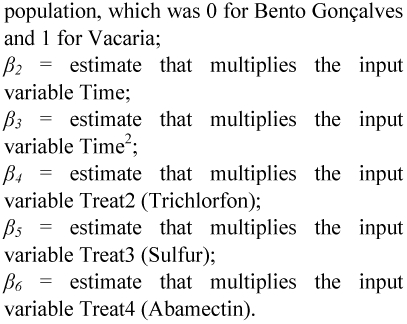

As an example, for the equation that gives female oviposition of *C. externa* from Bento Gonçalves treated with distilled water (control), the input variables Population, Treat2, Treat3, and Treat4 must have a value of 0.

**Table 1.**
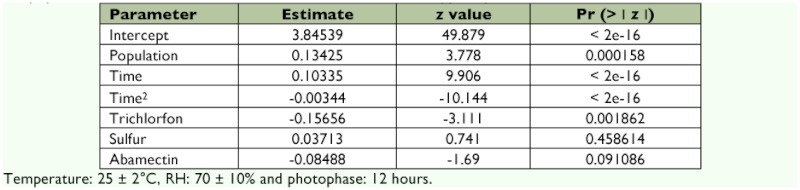
Parameter estimates used to calculate the equations leading to the oviposition model of *Chrysoperla externa* for the populations of Bento Gonçalves and Vacaria in relation to the applied pesticides.

### Ultrastructural analysis of *C. externa* eggs

Eggs laid by *C. externa* from both populations, treated with abamectin or sulfur, as well as distilled water (control), were prepared for later studies under scanning electronic microscopy, given the fact that these pesticides reduced viability rates through evaluation. Twenty newly laid eggs were used per treatment; they were transferred to plastic containers (Eppendorf, www.eppendorf.com) with capacities of 2.0 ml and subjected to a protocol for biological sampling preparation, according to the laboratory's routine techniques described by Borém et al. (2008). Then, the samples were studied under a scanning electronic microscope (LEO Evo40 XVP).

## Results

Six hours after the application of the pesticides, no compound had caused the death of any *C. externa*. However, 12 hours after application of carbaryl, fenitrothion, and methidathion significant mortality was observed in adults from both populations, and this situation remained unchanged until the last evaluation (120 hours after the beginning of the bioassay) when these compounds had caused the death of 100% of the individuals. Sulfur and abamectin also caused mortality of 6.7% and 10%, respectively, in adults from the Bento Gonçalves population until the end of the evaluations and were innocuous to those from Vacaria. Trichlorfon was harmless to adults of both populations, and trichlorfon and sulfur did not change the mortality pattern of any population throughout evaluation process (Table 2).

**Figure 1.**
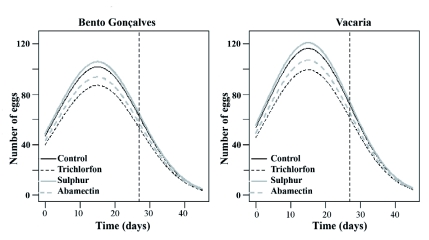
Oviposition estimates of *Chrysoperla externa* females from Bento Gonçalves and Vacaria, in function of pesticides application. High quality figures are available online.

Oviposition capacity of surviving *C. externa* treated with trichlorfon, sulfur, or abamectin was not reduced by these compounds in either of the studied populations. However, females from Bento Gonçalves treated with sulfur or abamectin showed similar variations in the mean amount of laid eggs throughout the evaluation period. Females from Vacaria had similar variations when treated with trichlorfon or sulfur (Table 3).

It was also verified that the peak of oviposition for all treatments happened near the 15th day after the beginning of oviposition, regardless of the population. The mean amount of eggs varied from 101.5 to 120.2 for females from Bento Gonçalves and from 124.8 to 142.8 for females from Vacaria, with nearly 40 eggs each day.

Oviposition capacity of *C. externa* was reduced for both populations. In all evaluated treatments from the 27th day of oviposition, oviposition capacity varied from 47.5 to 63.0 eggs per female for the Bento Gonçalves population, and from 47.8 to 60.7 for the Vacaria population (Table 3).

**Table 2.**
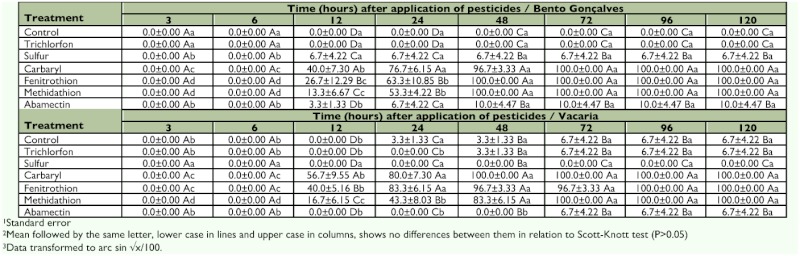
Accumulated mortality (± SE^1^) of *Chrysoperla externa* adults from Bento Gonçalves and Vacaria, Rio Grande do Sul — Brazil, until 120 hours after application of the pesticides^2,3^.

**Table 3.**
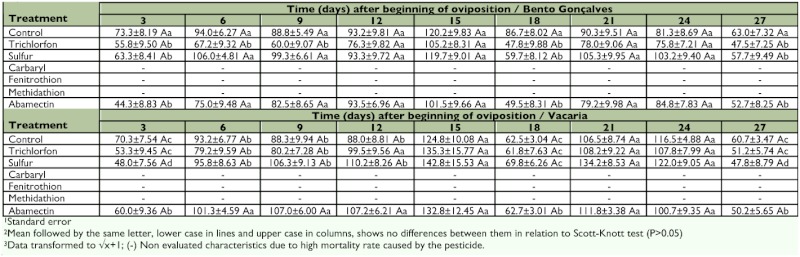
Number (± SE^1^) of eggs laid by *Chrysoperla externa* from Bento Gonçalves and Vacaria, Rio Grande do Sul — Brazil, treated with pesticides, throughout the 27 days of oviposition^2,3^.

**Table 4.**
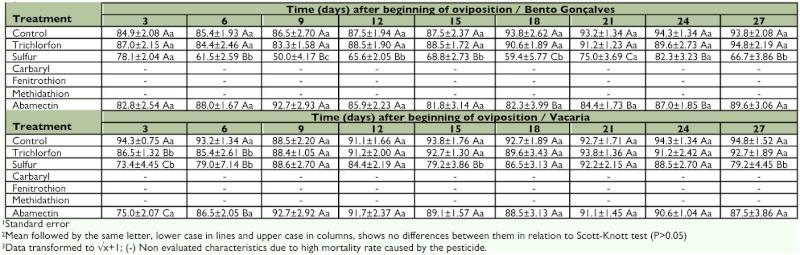
Viability (%) (± SE^1^) of eggs laid by *Chrysoperla externa* from Bento Gonçalves and Vacaria, Rio Grande do Sul - Brazil, treated with pesticides, throughout 27 days of oviposition^2^.

**Table 5.**
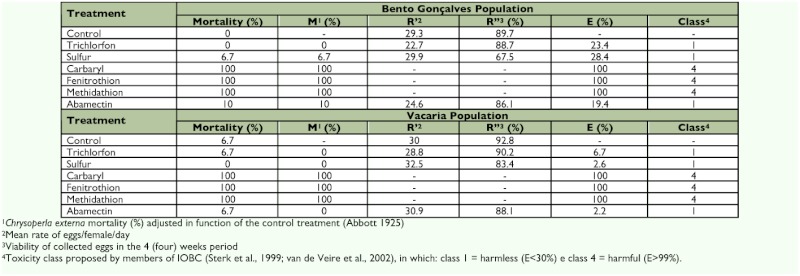
Effect of six different pesticides applied on *Chrysoperla externa* from Bento Gonçalves and Vacaria, in relation to adult mortality, oviposition capacity and viability of eggs from surviving females, total effect (E%) and toxicity classification.

Analysis of the data to develop a model that fit the obtained data and generation of equations that aim to predict *C. externa* oviposition from both studied populations evidenced that negative binomial (Pascal) was the best fit distribution with an AIC of 4175.1 and ratio between deviance and degrees of freedom of the residue equal to 443.55/425 (the result is 1.04), considered adequate by the residue analysis.

Oviposition modeling (Figure 1) showed that trichlorfon, followed by abamectin, were the most harmful compounds. These compounds affected oviposition of *C. externa* regardless of the origin of the studied *C. externa* population. Sulfur allowed the most oviposition with the mean varying around 50 to 100 eggs every three days for females from Bento Gonçalves and around 56 to 120 eggs for females from Vacaria. Oviposition behavior for females treated with the different pesticides was similar for both populations.

**Figure 2.**
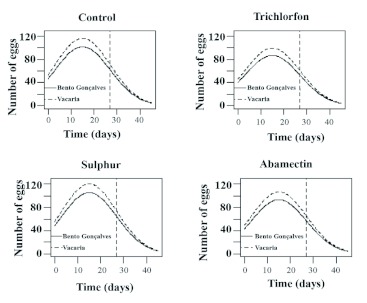
Oviposition estimates of *Chrysoperla externa* females from Bento Gonçalves and Vacaria, subjected to distilled water (control), trichlorfon, sulfur, or abamectin application. High quality figures are available online.

It was also observed that the *C. externa* oviposition estimates for each of the tested pesticides, and the control showed greater oviposition capacity for females from Vacaria. This was true for both obtained and predicted values (Figure 2).

Nevertheless, there was a trend that the mean amount of *C. externa* eggs laid, irrespective of the pesticide used, was equal at the end of the oviposition period for both populations based on the prediction made by the adjusted model (Figures 1 and 2).

As for egg viability, it was observed that sulfur was the most damaging to both *C. externa* populations. For *C. externa* from Bento Gonçalves, oviposition was reduced in every single evaluation except for the first. Changes in hatching eggs caused by sulfur were also observed through the evaluations varying from 50% to 82% for *C. externa* from Bento Gonçalves and from 73% to 92% for *C. externa* from Vacaria (Table 4).

**Figure 3.**
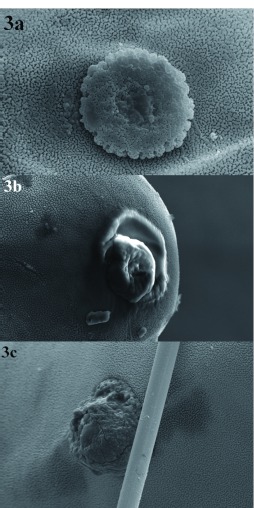
External morphology of the chorion and micropyle of *Chrysoperla externa* eggs from Bento Gonçalves, treated with distilled water (a), abamectin (b), or sulfur (c). High quality figures are available online.

Abamectin also negatively affected this biological parameter but just on the 18th, 21st, and 24th day after oviposition began for *C. externa* from Bento Gonçalves and on the first and second evaluations performed three and six days after oviposition began for the Vacaria population. In the other evaluations no differences were observed between abamectin and the control. Throughout the performed evaluations, no changes were verified in egg viability laid by *C. externa* treated with abamectin, regardless of the population (Table 4).

**Figure 4.**
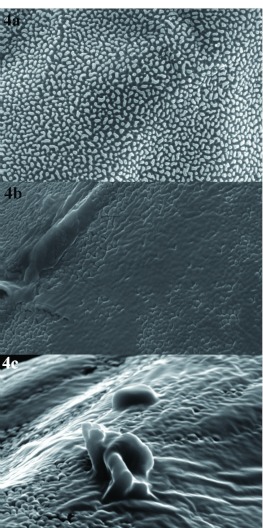
External surface of the chorion of *Chrysoperla externa* eggs from Bento Gonçalves, treated with distilled water (a), abamectin (b), or sulfur (c). High quality figures are available online.

Trichlorfon showed to be innocuous to *C. externa*, causing no reduction in viability of the eggs laid by treated females irrespective of the day of evaluation and irrespective of the studied population. Exceptions occurred in evaluations performed three and six days after the beginning of oviposition for *C. externa* from Vacaria when this pesticide provided egg viability of 86.5% and 85.4%, respectively (Table 4).

Based on the mortality caused by the compounds tested on *C. externa* from Bento Gonçalves and Vacaria and its effects on the reproductive capacity and egg viability (Tables 2, 3, and 4), trichlorfon, sulfur, and abamectin were classified as harmless (class 1), while carbaryl, fenitrothion, and methidathion were classified as harmful (class 4) for both of the studied populations (Table 5).

**Figure 5.**
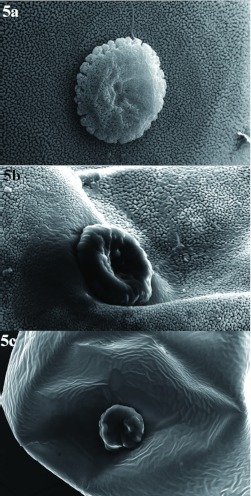
External morphology of the chorion and micropyle of *Chrysoperla externa* eggs from Vacaria, treated with distilled water (a), abamectin (b), or sulfur (c). High quality figures are available online.

**Figure 6.**
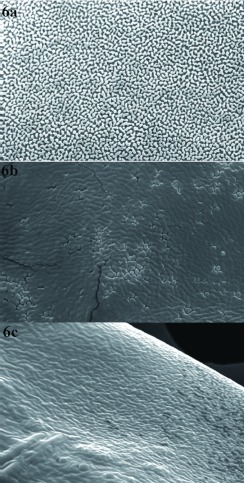
External surface of the chorion of *Chrysoperla externa* eggs from Vacaria, treated with distilled water (a), abamectin (b), or sulfur (c). High quality figures are available online.

Ultrastructural analysis of *C. externa* eggs from both populations treated with sulfur or abamectin, which negatively affected egg viability, showed that these compounds changed the chorion and micropyle morphology of the eggs compared to eggs from females treated with distilled water (Figures 3, 4, 5, and 6). The malformation occurrence frequencies in the samples observed under a scanning electron microscope were about 67% for eggs of *C. externa* treated with sulfur and nearly 50% for eggs laid by females treated with abamectin.

**Figure 7.**
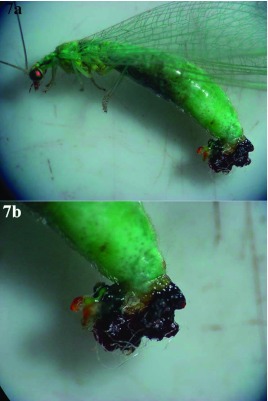
*Chrysoperla externa* female presenting deformations in the distal region of the abdomen and genitalia caused by the application of the fungicide sulfur (a); affected region in detail (b). High quality figures are available online.

It was also verified that some females of *C. externa* from both populations treated with sulfur showed malformations in the distal region of the abdomen and genitalia with the presence of dark, unidentified material (Figure 7).

## Discussion

The results for abamectin in the present research are similar to the outcome of Godoy et al. (2004), who also observed no significant differences in mortality rates between this compound and the control samples of *C. externa*.

The safety of sulfur on adult *C. externa* is related to the innate tolerance of this predator to acaricides and fungicides containing sulfur, since according Croft (1990), these compounds are considered selective to natural enemies.

Trichlorfon was observed to be innocuous to adult *C. externa*. This is possibly due to its inability to penetrate *C. externa*'s integument, as also related by Croft (1990) to *Chrysoperla cornea*. However, that author also commented that *C. externa* has developed low-level resistance to a wide range of conventional insecticides, including several organophosphates, carbamates, and some pyrethroids, and *C. externa* has widely adapted to the pesticide regimes used on apple trees. Detoxification factors can also provide selectivity to adults of *Chrysoperla* spp., as related to phosmet.

The results obtained in our research with carbaryl were also obtained by Wilkinson et al. (1975) and Güven and Göven (2005) for adults of *C. cornea* that resulted in 100% mortality, classifying carbaryl as harmful to this *Chrysoperla* species.

Results obtained in this study for carbaryl, fenitrothion, and methidathion, which caused 100% mortality, confirmed results achieved by Grafton-Cardwell and Hoy (1985), Singh and Varma (1986), and Mizell III and Schiffhauer (1990), who observed high susceptibility of *C. cornea* to carbamates and organophosphates. This shows the high toxicity of these pesticides to adults of several *Chysoperla* species, which may restrict its use in both integrated pest management programs and integrated Brazilian apple production.

Studies conducted by Vogt et al. (2001) and Bozsik et al. (2002) with *C. cornea* evidenced that carbaryl and malaoxon showed high inhibitory capacity on the acetylcolinesterase enzyme in this species, which also occurred in the present study with carbaryl, fenitrothion, and methidathion. The authors describe that acetylcolinesterase activity prediction appears to be an important tool for measuring differences either in susceptibility or tolerance of the species or in populations of a common enemy species in relation to potential side effects of a pesticide to the environment.

As for the reproductive capability of treated *C. externa* females, it was verified that the highest oviposition values achieved in this study were similar to those obtained by Ru et al. (1975) in studies about the biology of *C. externa*. It is believed that the Vacaria population presents greater reproductive potential when compared to the population from Bento Gonçalves, which must be considered when making use of *C. externa* in integrated pest management programs and in integrated apple production in southern Brazil. Probably the *C. externa* population from Vacaria is more fit because it has been regularly exposed to the evaluated pesticides before being tested in the laboratory. This population may have developed more tolerance (or resistance) to these pesticides than the Bento Gonçalves population, which has not been exposed to pesticides and has not developed resistance.

*C. externa* oviposition estimates (Figure 1) were based on the evaluations performed up to 27 days after the beginning of oviposition (dashed vertical line); hence the values are obtained from the prediction given by the adjusted model. Future research should consider a wider oviposition period. For example, during six or seven weeks, since the studies of this species (Núñez 1988; Carvalho et al. 1998; Silva et al. 2004) have been evidencing that the oviposition period can reach up to 100 days depending on food given to adults. Some researchers have already shown the possibility of evaluating *C. externa* oviposition subjected to pesticide application through selectivity tests for up to 50 days (Bueno and Freitas 2004).

Viability reductions, as observed mostly in eggs from *C. externa* treated with sulfur, may be a side effect of this pesticide on oogenesis, possibly on trophocytes (sister-cells of the oocytes) and responsible for their nutrition. According to Chapman (1998), the trophocytes malformation or the absorption of contaminated proteins by these cells may result in lack of nutrients for embryos or changes in embryo development, leading to embryo death. In this way, pesticides must have affected such physiological events and caused a reduction in viability rates for treated eggs.

The obtained toxicity classification for sulfur in this research confirmed the research of Silva et al. (2006) for adult *C. externa*. Silva et al. (2006) considered sulfur harmless to *C. externa* with total effect (E) lower than 30%. This result also matches those of Hassan et al. (1983, 1987, 1994) for the species *C. carnea*.

Silva et al. (2006) classified chlorpyrifos as harmful (class 4); this was the same classification given to fenitrothion and methidathion in the present study. Fenitrothion and methidathion are pesticides of the same chemical group of chlorpyrifos (organophosphates), which demonstrates the high toxicity of these compounds to *C. externa*.

Research conducted by Hassan et al. (1983, 1987) with *C. carnea* on the toxicity classifications attributed to trichlorfon, carbaryl, fenitrothion, and methidathion were the same classifications given to the same compounds in this study on *C. externa*. The methods used were identical.

The observed changes in the external surface of the chorion of eggs from females exposed to sulfur or abamectin residues suggests that the changes might have been induced by changes in the folicular cells responsible for the secretion of chorion layers, since shape modifications caused in the above mentioned cells are reflected in the chorion morphology (Chapman 1998). However, changes in the cells' constitution may also be responsible for modification in the chorion surface since the proteins synthesized by folicular cells behave as basic material to the chorion formation. These proteins also may affect the formation of aeropyle, micropyle, and other chorion pores.

It is believed that the abnormalities caused by sulfur and abamectin to both chorion and micropyle of eggs from treated *C. externa* may be responsible for the observed reduction of egg viability. According to Mazzini (1976) and Chapman (1998), alterations in any of the chorion layers may affect its permeability, and consequently, the loss of water, embryonic development, and egg viability. Still according to the same authors, abnormalities in cellular processes which are responsible for the micropyle formation may inhibit access for the sperm to the inner side of the egg and interfere in its fertilization and viability.

The causes of observed deformation at both the distal region of the abdomen and the genitalia of *C. externa* females from Bento Gonçalves and Vacaria treated with sulfur could not be explained by this research or found in scientific literature.

In conclusion, sulfur and abamectin are responsible for anomalies in the chorion and micropyle of *C. externa* eggs. Sulfur causes malformations in the genitalia of treated females. Sulfur, trichlorfon, and abamectin are harmless, whereas carbaryl, fenitrothion, and methidathion are harmful to adults of both studied populations, according to the IOBC toxicity classification.

## Abbreviation

IOBC: International Organization for Biological Control
